# Food Acquisition, Hygiene, and Generation of Domestic Waste in an Academic Community during the COVID-19 Pandemic

**DOI:** 10.3390/foods11233919

**Published:** 2022-12-05

**Authors:** Aldiane de Assis Costa, Bruna Leal Lima Maciel, Dirce Maria Marchioni, Priscilla Moura Rolim

**Affiliations:** 1 Department of Nutrition, Postgraduate Program in Nutrition, Center for Health Sciences, Federal University of Rio Grande do Norte, Natal 59075-000, RN, Brazil; 2 Nutrition Department, School of Public Health, University of São Paulo, São Paulo 01246-904, SP, Brazil

**Keywords:** food purchase, sanitization, waste, COVID-19, academic community

## Abstract

In 2020, the World Health Organization (WHO) declared COVID-19 a pandemic, and the closure of universities as a measure to prevent contamination directly affected academic communities. Access to food, though a basic need and a human right, was seriously affected. This study evaluated the locations and frequency of food acquisition; hand, food, and packaging hygiene habits; and household waste generation in an academic community during the COVID-19 pandemic. The research was cross-sectional and performed through an online questionnaire. Data (*n* = 1472) were analyzed using descriptive statistics; statistical tests were also applied, and *p* values < 0.01 were considered significant. Most of the population continued to purchase food in supermarkets (89.5%). The frequency of product orders from markets by delivery placed by professors and graduate students was also verified (31.7% and 24.2%). There was an increase in packaging hygiene in the studied population, as well as in fruit and vegetable hygiene; however, use of inappropriate methods was noted. This paper highlights important data on the behavior of an academic community dealing with the problem of solid waste generation during the pandemic. Moreover, there were no changes in waste generation during the pandemic, although there was an increase in packaging consumption (44%). Identifying the behavior of the university community regarding hygiene and food acquisition can help societies from the perspective of transforming habits related to food. Therefore, this research provides support for future investigations and interventions in the field of foods and post-pandemic sustainability.

## 1. Introduction

In December 2019, a severe acute respiratory syndrome caused by coronavirus 2 (SARS-CoV-2) created a pandemic without precedent, posing a threat to the health of the world’s population [1,2]. In the academic sphere, in addition to all the employees that make up the academic community, it is estimated that 8.5 million university students were affected by the closing of universities [3]. Furthermore, with university activities suspended, including university restaurants, the academic community, the members of which most likely had their meals at the university or nearby, began to eat at home [4,5].

During this global health crisis, negative socioeconomic impacts affected the population, especially individuals in more vulnerable social conditions. The health crisis and its necessary prevention measures generated great difficulties, as well as demand for food business alternatives, leading the food sector to integrate the digital food environment through food delivery apps [5].

Food and nutrition security, food security, and sustainability are dimensions of the food system, and they were strongly affected during the pandemic [6]. Imposed by the pandemic scenario, changes in the way foods were purchased occurred, such as increases in purchases via delivery and in the preparation of meals at home. The impacts on environmental sustainability were also worrying, primarily due to potential increases in waste generation in the household environment resulting from the greater use of disposable packaging [7].

Questions also arose regarding the transmission of COVID-19 through food and the contamination of surfaces and packaging. To date, there are no confirmed cases of transmission through food or records in the literature involving food in epidemics caused by other coronaviruses. However, the risks could not be denied [8,9]. Therefore, national and international recommendations regarding correct hand hygiene, working environments, contact surfaces, and correct food hygiene have been highlighted [10,11].

This paper discusses some of the perspectives on this topic. Although there is a lot of research on the subject, as well as many campaigns about the importance of hand and food hygiene, the population in general still lacks knowledge about the field, especially regarding the use of chemicals. Issues such as food hygiene and packaging at home should be studied more in the post-pandemic period, especially in relation to foodborne diseases, to ensure safer food. Another question concerns consumers’ behavior regarding food acquisition. The university community stands out as a relevant place for transformative practices for society, such as the adoption of healthy alternatives in the face of adversity, as in the case of the pandemic. The behavior mentioned above was related to the generation of household waste, such as food and packaging waste. There are few studies that have investigated the association between waste generation, pandemics and university communities.

To understand the impact of issues related to food practices and waste generation in the university community in the face of this unexpected and emergency pandemic situation, we investigated what changes occurred in food acquisition and hygiene. We also measured the environmental impacts of domestic waste generation in the academic population during the COVID-19 pandemic. The study aimed to evaluate food acquisition locations and frequency, hand hygiene habits, foods and packaging, and household waste generation in an academic community during the pandemic. 

## 2. Materials and Methods

Ethical approval: The study was reviewed and approved by the Ethics Committee of the Onofre Lopes University Hospital (CEP-HUOL) at the Federal University of Rio Grande do Norte (UFRN), number 35918620.7.0000.5292. All ethical precepts contained resolution no. 466 of the CNS from 2012 were followed [12].

Characterization of the study and study population: The study was descriptive, observational, and cross-sectional and was performed in the UFRN academic community, with data collection between September 2020 and February 2021. This study is part of the multicenter Brazuca COVID study investigating food insecurity, nutritional status, and lifestyle among students, teachers, and technical-administrative staff in the academic community during the COVID-19 pandemic.

The academic community at UFRN consists of 30,456 undergraduates, 14,337 graduate students, and 5403 technical-administrative staff and professors according to UFRN’s estimate [13,14]. The sample size was determined by convenience from the number of people who responded to the online form (*n* = 1472), and non-probabilistic sampling was used. A posteriori power analysis was performed, considering the sample size (*n* = 1472), using the chi-squared test (X^2^) in GPower software (Dusseldorf, Germany). The power achieved was 86%, assuming a small effect size at 0.10 and alpha at 0.05.

Data collection: The study used an online questionnaire for data collection. All active students, employees and professors with e-mail addresses registered in the UFRN system were included; participants under 18 years were excluded. Participants were invited by e-mail to participate in the research. Before sending the questionnaire, the research was publicized using social media to reveal its purposes and relevance, promoting adherence to the study. A link to a free and informed consent form was sent in the body of the email, which clarified the research objectives and methodology. After clicking on “I agree” to participate in the research, the participant received (by e-mail) a signed copy of the informed consent form (signed by the researcher responsible for the study). The online questionnaire was prepared using the Google Forms platform (Google Forms). The questionnaire consisted of 108 questions and the average estimated time for complete response was 20 min. 

Assessment of the socioeconomic profile and institutional position: The first block of questions served to outline socioeconomic profiles and the institutional position of the participant; it contained nine questions about employment, course, place of employment, sex, age, race, marital status, family income, and income changes during the pandemic period. The questions used pre-established answers, except for those referring to the educational course and location.

Assessment of food purchases: Assessment comprised one block with 1 question (how often have you purchased food to prepare your meals) and 11 response options for locations where food was purchased (free market, rural producer/family farming, grocery store/Hortifruti/grocery store/market-sourced fair, other fairs or organic producers, fruit and vegetable seller operating through phone/delivery/application, non-perishable food delivery through phone/mobile app, meal delivery through phone/mobile app, hyper-/supermarket, minimarket/grocery store, donations, at-home vegetable gardens or fruit trees), as well as a single pre-established response in relation to acquisition frequencies (0—never; 1—once week; 2—two to three times a week; 3—once a fortnight; and 4—once a month). 

Assessment of hand, food and packaging hygiene: This was evaluated through a block of ten questions related to the pandemic and the previous period with pre-established answers. Questions about the frequency and method of cleaning fruits, vegetables and packaging before and during the pandemic; about products used for hygiene; and concerning hand washing before preparing food were included. The products used in the process of cleaning fruits, vegetables and packaging were grouped into products for cleaning (soap and water or vinegar), disinfection (bleach, sodium hypochlorite, 70% alcohol) and cleaning together with disinfection (hydrogen peroxide).

Generation of domestic waste: The questionnaire contained five questions about domestic waste separation, the complete use of food, increases and decreases in food waste before and during the pandemic, use/consumption of packaging and food waste during the pandemic.

Statistical analysis: Descriptive analyses were performed, testing the data distribution normality using the Kolmogorov–Smirnov test to present data in the form of means or medians with relevant dispersion measures. Categorical variables were presented through frequency distribution, and associations were evaluated using the chi-squared test. For all tests performed, in order to avoid type 1 errors, *p* values lower than 0.01 were considered significant, given the large sample size. Data were downloaded and codified into a database using Microsoft Office Excel 2007^®.^ The statistical analysis was performed using the Statistical Package for Social Sciences version 11.5 (SPSS Inc., Chicago, IL, USA) and Graph Pad Prism version 3.0 (Graph Pad Software, San Diego, CA, USA).

## 3. Results

### 3.1. Characterization of the Studied Population

Data concerning the studied population are shown in Table 1.

Most of the studied individuals were female (65.8%), with a predominance of this gender in all studied academic positions. Young adults aged between 18 and 35 accounted for 71.4% of the sample, and the largest proportion (54.3%) self-reported being white, followed by brown (36.7%), which together accounted for more than 90% of the studied population. For marital status, most were single (64.9%). For family income, 30.0% received between one and three minimum wages, and 45.1% experienced changes in the family income during the pandemic.

### 3.2. Food Purchasing

The results for food purchase locations and the frequency of delivery orders for ready meals are presented in Table 2.

During the COVID-19 pandemic, supermarkets (89.5%), grocery stores (49.9%) and street markets (33.3%) were the locations mainly used by the research participants to buy food. Professors were the group who purchased the most food from organic fairs (17.4%) and by delivery from markets using phone/mobile apps (31.7%). Most of the studied population purchased ready-to-eat meals one to two days/week (27.3%) or almost never (22.9%). 

### 3.3. Hygiene Assessment

**Figure 1:**
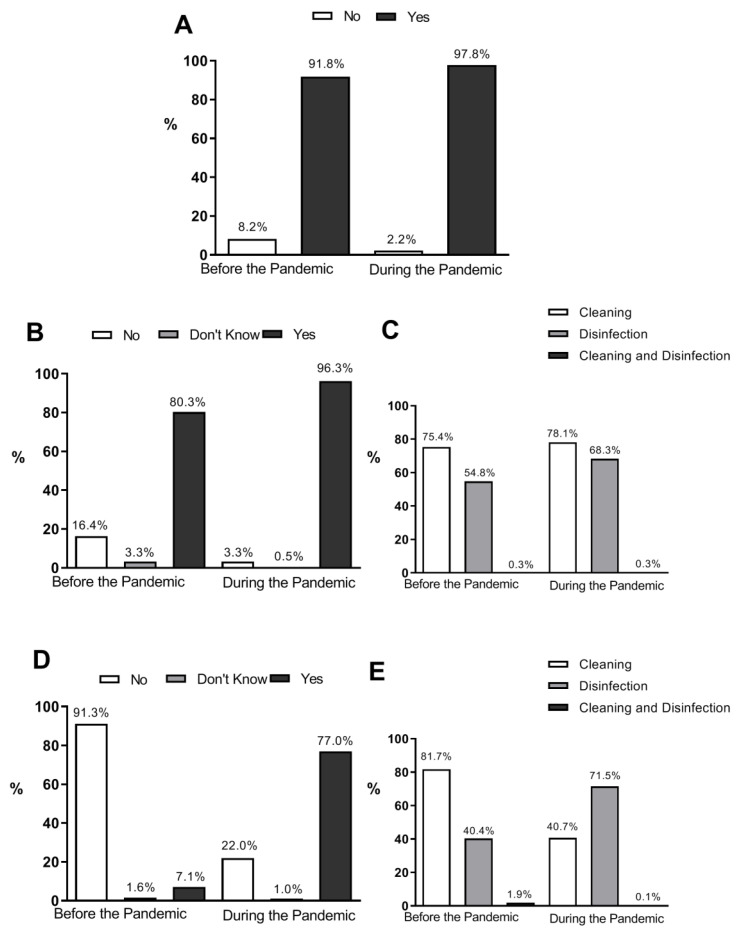
Frequency of hand washing, fruit/vegetable washing and packaging hygienization before and during the COVID-19 pandemic in an academic community (*n* = 1472). (**A**) Hand hygiene. (**B**) Cleaning of fruit/vegetables. (**C**) Types of products used for cleaning fruit/vegetables. (**D**) Hygienization of packaging. (**E**) Types of products used for cleaning packaging (chi-squared, *p* < 0.0001). Cleaning: soap and water, vinegar; disinfection: bleach, sodium hypochlorite, 70% alcohol; cleaning plus disinfection: sodium peroxide.

Data showed that 91.8% and 97.8% of the studied population reported the habit of washing hands before and during the pandemic, respectively. Thus, hand washing was a common practice among the studied population and was performed with even greater success during the pandemic (Figure 1A).

The cleaning of fruits and vegetables before the pandemic was carried out by 80.3% of the participants. This habit increased to 90.6% during the pandemic (Figure 1B). The participants mentioned using all the products in the questionnaire’s pre-established responses (water and soap, vinegar, bleach, 70% alcohol, sodium hypochlorite, and hydrogen peroxide). Despite the satisfactory results for hygiene, when investigating product usage, there was some inconsistency and lack of information about its correct use since, both before and during the pandemic, a high percentage (75.4% and 78.1%) of respondents used cleaning products. However, there was an increase from 54.8% to 68.3% among those who started using products for disinfection (Figure 1C).

A finding considered very relevant in the analysis regarding the food–COVID-19 nexus was that concerning packaging hygiene. The products mentioned were the same used for cleaning fruits and vegetables. Comparing packaging hygiene before the pandemic (7.1%) and after the pandemic (77.0%), there was a great increase in the practice (Figure 1D), as well as greater consistency in the use of the correct products. However, the predominance of cleaning products (81.7%) before and during the pandemic (40.7%) potentially negatively affected this practice.

When an association was developed between the variables of hand, food and packaging hygiene and the responses of the participants according to the areas of knowledge, the results showed that, during the pandemic, individuals involved in health sciences cleaned packaging more (84.6%) than those involved in other areas (75%) (Table 3).

### 3.4. Assessment of Waste Generation 

Regarding data on household food waste (Table 4), the participants pointed out that there was no increase (53.7%) or there was a decrease (43%) in household food waste. However, 44.9% of the studied academic community reported an increase in packaging consumption. On the other hand, most of the studied population (59.5%) reported not separating domestic waste into organic and inorganic waste and not producing fertilizer through composting (79.8%). 

## 4. Discussion

Changes in eating habits occur in different contexts influenced by globalized markets, changes in lifestyles and increased consumption outside the home [15]. Further, concerns about the sustainability of the planet [16], food quality, and food risk also affect eating habits [17]. 

Many of these changes were felt during the COVID-19 pandemic. Amid the restrictions of social isolation, the practice of food shopping continued to be carried out in person by most consumers, and the most sought-after locations for food purchasing were those presenting a greater variety of options, such as supermarkets in their various types, and those that different financial conditions [18]. These were still considered safer during the pandemic period by 70% of consumers [19]. Grocery stores and street markets followed on the list of preferences, suggesting that ease, proximity and prices remained attractive [20].

Other food acquisition locations, such as organic fairs and market purchases through delivery apps, deserve attention. In our study, professors used delivery apps more frequently, showing notable data for two contributing factors, higher income and higher education level, which reflected more comprehensive access to new technologies. In line with our findings, Costa et al. (2022), in a study carried out in Fortaleza (Brazil), outlined a profile in relation to consumers at organic fairs, and similar results were also found by Zamberlan et al. (2017) [21,22]. A study by Xie et al. (2020) in China concluded that the COVID-19 crisis had a positive impact on the attitudes of respondents towards organic food, encouraging this type of agriculture [23].

In addition to the increase in market purchases via delivery during the pandemic, innovative ready-to-eat delivery formats managed to grow, using the virtual environment as a tool for selling food [24]. The ready-to-eat delivery practice was mainly chosen twice a week by professors and graduate students. This result reinforces the purchasing power factor, since it presents a positive relationship with income and an inverse relationship with age. This points to jovial behavior—similar to the findings from the Instituto QualiBest (2021) and Conde (2022), which highlighted the frequency of this trend on weekends [25,26]—and leads to a potential hypothesis that the percentage of people who have never bought food using this tool is small and related to a lack of purchasing power, and/or fear of contamination, since COVID-19 is transmitted from person to person.

Purchasing food via delivery has increased due to behavioral trends, such as the digitalization of businesses, the use of digital channels of interaction with consumers and the feeling of security in making purchases through digital means [27]. The EY Future Consumer Index survey showed that the food segment presented a 62% drop in visits to physical stores and a 32% growth in online purchases, behaviors already noticed and pointed out by Nielsen (2020) since the beginning of the pandemic. Online purchases helped many establishments avoid bankruptcy [19,28].

Both in-person and online, food purchases caused concerns regarding possible contamination by the virus that causes COVID-19, and after acquiring the food, these feelings involved hygiene.

Hand hygiene during the pandemic was seen as a coping measure to reduce the chances of transmission, and it was necessarily maintained and did not fall into disuse [29]. A study by Gonçalves and Toriani (2021) provided data similar to the present study, showing that 94.2% performed the practice. However, Dalmolin et al. (2021) reported a divergence, showing that only 52.1% of respondents washed their hands during the pandemic [30,31].

Promotion of hand hygiene should be considered a priority awareness investment, and not only in the current situation, since it is effective behavior in preventing the transmission of disease, it is easy to perform and it has little cost [32]. It is important to highlight the impacts on human health and the risks of contamination when using delivery, mainly because, during the delivery of an order, there is contact with the delivery person, who is a potential vector of the virus. Practices must be devised to minimize this risk [33]. 

In addition to hand hygiene, due to certain surfaces, such as plastic, cardboard and styrofoam, being transmission channels (as they allow the virus to remain for hours or days), hygiene needs to be incorporated into the handling of packaging in the domestic routine [34]. A significant increase in the practice of sanitizing packaging has also been noticed in other studies. Before the pandemic, 68.3% of those interviewed by Gonçalves and Toriani (2021) performed the sanitization of food and packaging. This rose to 74.2% at the beginning of the pandemic. However, despite good information, this percentage dropped in just over three months, showing the importance of continuing to highlight the evils arising from a lack of packaging hygiene through public policies [35]. 

Therefore, food hygiene—especially for fruit/vegetables, which are most often consumed raw—and packaging hygiene, which gained notoriety in the pandemic, need to be better disseminated among the population. The lack of information about correct hygiene can lead to inefficacies. For example, it is only possible to clean using the proper sanitizer. Damolin et al. (2021) showed that 64.2% of their study population only used water to clean vegetables, and another portion used the wrong products [29]. These data corroborate those found in the present study.

As a measure to combat COVID-19, chemical sanitizing agents were recommended and made available to the population. Seventy-percent alcohol was the most widespread and commonly used product at the beginning of the pandemic, and bleach and sodium hypochlorite were the most accessible products, being suggested as alternatives by the WHO [35].

Due to the changes in food acquisition and hygiene, a higher percentage of product containers were used by households. Thus, domestic waste generation also demonstrated important changes during the pandemic. From this perspective, solid waste management, which is already a public health problem in many countries around the world, developed as another problem needing palliative solutions to avoid the negative socio-environmental effects brought by new habits [36]. On the other hand, the need to improve management of both collection and disposal of these materials, and the risk of contamination through them, has encouraged many countries to partially or totally suspend collection of domestic waste [37,38,39,40].

According to Teixeira and Mourão (2021), most people who use delivery services discard the packages involved in the order, although a minority use them for some other purpose [41]. As commerce in food and market applications further expanded in the first half of 2020—when, in Brazil, 22 million cell phones were already using a delivery application—this way of purchasing food has impacted organic waste generation through an increase in the use of disposable packaging, who presented results showing that, more than 80% of the time, food comes with more than one package [42].

The behavior report led the ABRELPE (2020) to estimate that the increase in domestic waste generation in Brazil would grow from around 20% to 25% [43], which was confirmed in the data from the present study regarding packaging consumption. Higher values were recorded in Malaysia in the early days of the pandemic, where an increase in food waste was also observed. This finding differs from our research, suggesting that participants in the academic community had no accurate perception of how much waste they produced, probably because, before the pandemic, this population consumed meals outside the home [44].

Despite being considered economically and socially important for generating more jobs and ensuring accessibility to food, as well as having been incorporated by decree as an essential activity in the pandemic period, this segment presents severe environmental problems due to the increase in packaging, especially plastic [45,46,47,48].

In addition to packaging, the increase in household food waste has led many countries to rethink the logistics of public household waste collection. Before the pandemic, when waste separation and disposal did not occur satisfactorily, short-term palliative suggestions, such as the mixed collection of all solid waste destined for landfills and incineration, caused concern, being seen as a setback. These measures went against strategies already outlined by countries. Thus, researchers and policymakers must work to raise awareness about selective collection and appeal for reductions, recycling and reuse worldwide [40,49,50,51,52].

As a limitation of this study, the online data collection might have limited the research to those with access to and familiarity with technologies and digital media. However, by the time of data collection, the university was already conducting online (remote) classes, and those vulnerable students who requested internet assistance received it. The non-probabilistic sampling may have led to a selection bias in the motivation to answer the questionnaire, which could have been higher among those who felt more affected by the pandemic. However, studying those more affected was also within the scope of the research. 

In addition to being innovative, the strength of this study was its investigation of the locations and frequency of food acquisition; hand, food and packaging hygiene habits; and household waste generation in the broader academic community (undergraduates, graduate students, technical-administrative staff and professors) at the time of the COVID-19 pandemic. In addition, the investigated aspects are essential to understanding an academic community’s behavior in times of isolation due to a pandemic. Thus, the results can contribute to the design of public policy strategies for this population. Moreover, waste management is a problem that needs to be urgently addressed through the adoption of easy-to-implement measures. Therefore, the participation of public authorities and the population as active contributors is fundamental, and public policies should also address this.

## 5. Conclusions

In the studied academic community, food purchases were preferentially carried out in supermarkets during the pandemic. Practices of hand hygiene were intensified in the pandemic period, as well as fruit/vegetable hygiene. Packaging hygiene, which was not practiced previously, increased during the pandemic, but there were difficulties in choosing the correct product. Food waste was little noticed by the survey respondents; however, an increase in packaging in households was noted. Most of the studied population did not separate household waste and did not preserve food waste for reuse. Given our results, further research is needed to implement food, hygiene and sustainability strategies to control and alleviate the continuing pandemic among the studied academic community. Our results might help the programming of public policies directed toward the academic community, which are still lacking in Brazil.

## Figures and Tables

**Table 1:** Socioeconomic characterization of the studied population according to institutional positions (*n* = 1472).

Variables	Total	Undergraduates	Graduate Students	Technical-Administrative Staff	Professors	*p* Value
	*n* (%)	*n* (%)	*n* (%)	*n* (%)	*n* (%)	
Sex						
Male	503 (34.2)	317 (35.0)	62 (25.0)	60 (38.5)	64 (39.8)	0.005
Female	969 (65.8)	590 (65.0)	186 (75.0)	96 (61.5)	97 (60.2)	
Age (years)						
18–35	1050 (71.4)	774 (73.7)	189 (18.0)	61 (5.8)	26 (2.5)	0.000
36–50	292 (19.8)	102 (34.9)	53 (18.1)	60 (20.6)	77 (26.4)	
51–65	112 (7.6)	28 (25.0)	6 (5.4)	32 (28.6)	46 (41.0)	
>65	18 (1.2)	3 (16.7)	0 (0.0)	3 (16.7)	12 (66.7)	
Race						
Asian	6 (0.4)	4 (0.4)	2 (0.8)	0 (0.0)	0 (0.0)	0.011
White	800 (54.3)	457 (50.4)	145 (58.5)	91 (58.3)	107 (66.5)	
Indigenous	6 (0.4)	2 (0.2)	1 (0.4)	01 (0.6)	2 (1.2)	
Brown	540 (36.7)	360 (39.7)	82 (33.1)	55 (35.3)	43 (26.7)	
Black	120 (8.2)	84 (9.3)	18 (7.3)	09 (5.8)	9 (5.6)	
Marital status						
Single	956 (64.9)	725 (79.9)	146 (58.9)	52 (33.3)	33 (20.5)	0.000
Married	322 (21.9)	93 (10.3)	66 (26.6)	75 (48.1)	88 (54.7)	
Stable union	135 (9.2)	61 (6.7)	32 (12.9)	22 (14.1)	20 (12.4)	
Divorced	52 (3.5)	27 (3.0)	3 (1.2)	5 (3.2)	17 (10.6)	
Widower	7 (0.5)	1 (0.1)	1 (0.4)	2 (1.3)	3 (1.9)	
Family income (BRL)						
No income	52 (3.5)	48 (5.3)	4 (1.6)	0 (0.0)	0 (0.0)	0.000
≤1100.00	214 (14.5)	204 (22.5)	10 (4.0)	0 (0.0)	0 (0.0)	
1101.00 to 3300.00	441 (30.0)	335 (36.9)	93 (37.5)	12 (7.7)	1 (0.6)	
3301.00 to 6600.00	301 (20.4)	183 (20.2)	62 (25.0)	48 (30.8)	8 (5.0)	
6601.00 to 9900.00	184 (12.5)	63 (6.9)	41 (16.5)	45 (28.8)	35 (21.7)	
9901.00 to 13,200.00	126 (8.6)	29 (3.2)	23 (9.3)	30 (19.2)	44 (27.3)	
13,201.00 to 16,500.00	61 (4.1)	19 (2.1)	6 (2.4)	15 (9.6)	21 (13.0)	
>16,500.00	93 (6.3)	26 (2.9)	9 (3.6)	06 (3.8)	52 (32.3)	
Income change during the pandemic						
No	658 (44.7)	343 (37.8)	110 (44.4)	98 (62.8)	107 (66.5)	0.000
Yes, increase	150 (10.2)	107 (11.8)	32 (12.9)	6 (3.8)	5 (3.1)	
Yes, decrease	664 (45.1)	457 (50.4)	106 (42.7)	52 (33.3)	49 (30.4)	

Statistical significance: *p* < 0.01. *n* = 1472.

**Table 2:** Food purchase locations and frequency of delivery orders for ready meals during the COVID-19 pandemic in an academic community (*n* = 1472).

Locations	Total	Undergraduates	Graduate Students	Technical-Administrative Staff	Professors	*p* Value
	*n* (%)	*n* (%)	*n* (%)	*n* (%)	*n* (%)	
Open fair	490 (33.3)	355 (39.1)	78 (31.5)	33 (21.2)	24 (14.9)	0.000
Rural producer	167 (11.3)	102 (11.02)	30 (12.1)	8 (5.1)	27 (16.8)	0.013
Street market	328 (22.3)	217 (23.9)	52 (21.0)	23 (14.7)	36 (22.4)	0.079
Organic fair	94 (6.4)	39 (4.3)	15 (6.0)	12 (7.7)	28 (17.4)	0.000
Vegetables by delivery	160 (10.9)	66 (7.3)	34 (13.7)	21 (13.5)	39 (24.2)	0.000
Delivery by phone/mobile app	329 (22.4)	181 (20.0)	60 (24.2)	37 (23.7)	51 (31.7)	0.008
Supermarket	1318 (89.5)	811 (89.4)	224 (90.3)	147 (94.2)	136 (84.5)	0.041
Grocery stores	734 (49.9)	518 (57.1)	107 (43.1)	56 (35.9)	53 (32.9)	0.000
Donations	104 (7.1)	93 (10.3)	7 (2.8)	2 (1.3)	2 (1.2)	0.000
At home gardens/trees	172 (11.7)	117 (12.9)	30 (12.1)	11 (7.1)	14 (8.7)	0.113
Other	59 (4.0)	36 (4.0)	4 (1.6)	11 (7.1)	08 (5.0)	0.049
Frequency of orders for delivery of ready-to-eat meals						
1–2 days/week	401 (27.3)	212 (23.4)	89 (35.9)	45 (29.0)	55 (34.2)	0.000
3–4 days/week	134 (9.1)	65 (7.2)	32 (12.9)	19 (12.3)	18 (11.2)	
5–6 days/week	22 (1.5)	15 (1.7)	01 (0.4)	2 (1.3)	4 (2.5)	
Daily	16 (1.1)	5 (0.6)	0 (0.0)	6 (3.9)	5 (3.1)	
2 days/week	253 (17.2)	153 (16.9)	49 (19.8)	25 (16.1)	26 (16.1)	
Monthly	142 (9.7)	98 (10.4)	16 (6.5)	18 (11.6)	10 (6.2)	
Almost never	337 (22.9)	237 (26.2)	43 (17.3)	27 (17.4)	30 (18.6)	
Never	166 (11.3)	122 (13.5)	18 (7.3)	13 (8.4)	13 (8.1)	

Statistical significance: *p* < 0.01.

**Table 3:** Performance of hand washing, fruit/vegetable washing and packaging hygienization before and during the COVID-19 pandemic according to knowledge area (*n* = 1472).

Variables	Total	Health Sciences	Other Areas *	*p* Value
	*n* (%)	*n* (%)	*n* (%)	
Performing hand washing before the pandemic	1279 (86.8)	255 (93.7)	1024 (91.6)	0.143
Cleaning of fruit and vegetables before the pandemic	1118 (80.5)	222 (81.6)	896 (80.2)	0.516
Cleaning of packaging before the pandemic	98 (7.1)	18 (6.6)	80 (7.2)	0.638
Performing hand washing during the pandemic	1358 (97.8)	269 (98.9)	1089 (97.5)	0.160
Cleaning of fruit and vegetables during the pandemic	1337 (96.3)	266 (97.8)	1071 (95.9)	0.323
Cleaning of packaging during the pandemic	1068 (76.9)	230 (84.6)	838 (75.0)	0.003

Statistical significance: *p* < 0.01. * Humanities and Technology.

**Table 4:** Generation and management of household food waste during the pandemic in an academic community (*n* = 1472).

Variables	Total	Undergraduates	Graduate Students	Technical-Administrative Staff	Professors	*p* Value
	*n* (%)	*n* (%)	*n* (%)	*n* (%)	*n* (%)	
During the pandemic, was there an increase or decrease in food waste?							
Increased	150 (10.2)	86 (9.5)	32 (12.9)	19 (12.2)	13 (8.1)	0.065
Decreased	469 (31.9)	305 (33.6)	63 (25.4)	51 (32.7)	50 (31.1)	
No change	790 (53.7)	472 (52.0)	141 (56.9)	80 (51.3)	97 (60.2)	
Do not know	63 (4.3)	44 (4.9)	12 (4.8)	6 (3.8)	1 (0.6)	
During the pandemic, was there an increase or decrease in the consumption of packaging?							
Increased	661 (44.9)	376 (41.5)	133 (53.6)	71 (45.5)	81 (50.3)	0.002
Decreased	141 (9.6)	91 (10.0)	21 (8.5)	17 (10.9)	12 (7.5)	
No change	570 (38.7)	361 (39.8)	82 (33.1)	62 (39.7)	65 (40.4)	
Do not know	100 (6.8)	79 (8.7)	12 (4.8)	6 (3.8)	3 (1.9)	
During the pandemic, was there an increase or decrease in the residues?							
Increased	528 (35.9)	296 (32.6)	98 (39.5)	67 (42.9)	67 (41.6)	0.001
Decreased	187 (12.7)	127 (14.0)	22 (8.9)	17 (10.9)	21 (13.0)	
No change	633 (43.0)	390 (43.0)	108 (43.5)	65 (41.7)	70 (43.5)	
Do not know	124 (8.4)	94 (10.4)	20 (8.1)	7 (4.5)	3 (1.9)	
Was domestic waste seperated into reusable and organic materials?							
No	876 (59.5)	579 (63.8)	157 (63.3)	74 (47.4)	66 (41.0)	0.000
Do not know	21 (1.4)	18 (2.0)	1 (0.4)	2 (1.3)	0 (0.0)	
Yes	575 (39.1)	310 (34.2)	90 (36.3)	80 (51.3)	95 (59.0)	
Was organic waste used for the production of fertilizer through composting?							
No	1174 (79.8)	691 (76.2)	209 (84.3)	140 (89.7)	134 (83.2)	0.000
Do not know	39 (2.6)	25 (2.8)	04 (1.6)	4 (2.6)	6 (3.7)	
Yes	259 (17.6)	191 (21.1)	35 (14.1)	12 (7.7)	21 (13.0)	

Statistical significance *p* < 0.01.

## Data Availability

The data presented in this study are available on request from the corresponding author.
